# Gold Linings and Strengtheners for Vulcanite Plates

**Published:** 1898-11

**Authors:** J. H. Gartrell


### GOLD LININGS AND STRENGTHENERS FOR VULCANITE PLATES.[1]

BY J. H. GARTRELL.

The subject of lining and strengthening vulcanite plates is one that I have thought of sufficient interest to bring before you. It has doubtless had more or less attention from all who have given much attention to vulcanite dentures. Various experiments have been made since vulcanite was first introduced with a view of

[1] Read at the Annual Meeting held in Bath.

developing some process by which a durable gold surface may be produced on that portion of the rubber denture which fits over the alveolar and palatal portion of the mouth.

It will, I think, be generally admitted by those who have observed the effects of vulcanite upon the gums for any length of time that it seriously produces undue absorption of the alveolar process. The cause is, without doubt, due to the non-conducting properties of rubber, and in consequence the retention of undue heat. Some writers on the subject have attributed the deleterious effects to mercury-poisoning, but vermilion in combination with rubber when worn in the mouth is unlikely to produce injurious effects. It is not probable that this compound in contact with saliva is chemically decomposed and converted into a poisonous salt of mercury. Almost the last case I had to deal with before leaving home was to make a new upper continuous gum set for a patient who had worn a continuous gum set for about twenty years. I have the model of the mouth here, from which you may see that very little absorption of the ridge can have taken place in all that time. If a vulcanite plate had been worn during such a period I should expect to find the ridge greatly absorbed. Metal strengtheners for vulcanite plates have been generally fitted to the lingual surface, probably by way of ornament as much as to strengthen the plate. I have a set here which illustrates this method of fitting the gold to the lingual surface. In this particular case it would seem to be unnecessary to strengthen the rubber with metal, seeing the plate weighs over two ounces. As the patient wore this plate with satisfaction for a number of years, it goes to prove that weight is not of so much importance as the fit.

The method that has been usually adopted for lining the palatal surface with gold is to use gold-foil pressed over the model before finally closing the flask. To increase the adhesion to the rubber a modification of this method has been introduced in the United States, and known as "vulcan gold lining." It is a pure gold sheet covered on one side with a thin coating of silver. The idea is that the sulphur in the rubber, acting upon the silver, produces a condition of surface favorable to adhesion. This combination has proved a failure in my hands, as the silver in vulcanizing combines completely with the sulphur in the rubber, forming silver sulphide, from which the gold is readily stripped with the finger-nail. Tinning the surface of the silver will prevent this action; but after many experiments I have adopted a sheet metal composed of pure gold on one side and an alloy of silver and platina on the other, similar to the metal known as dental alloy. The object of using platina in the alloy is to prevent the silver being converted to silver sulphide. This composite metal also differs from the American preparation in being much thicker and substantial, or a sheet metal of No. 2 gauge or No. 32 American gauge instead of a foil. It consequently requires to be swaged to form the palatal surface of a vulcanite set, and is a strengthener as well as a pure gold lining. The method of making this sheet metal is borrowed from the method of preparing the celebrated Sheffield silver plate, which is well known to be much stronger and more durable than electro-plating. A plate of pure gold is prepared, and another of the silver platina alloy; the two plates are placed together and heated to redness, when great pressure is put upon them in an hydraulic press. This process unites them into a solid plate, which is then rolled in a flatting mill to the required gauge. The proportions of gold to the silver platina alloy I use are one ounce of pure gold to four ounces of the alloy. This gives a strong and durable coating of gold, which retains the color in the mouth under all conditions; 22-carat gold has been tried, but oxidation of the copper in the alloy prevented adhesion with silver; aluminum and gold will not combine for the same reason.

To prepare a plate of this composite metal for lining a vulcanite set, a thin metal model is made, either by casting fusible metal into a plaster impression or by moulding a plaster model in sand and obtaining a casting in fusible metal, die metal, tin, or zinc. The first and second metals are preferable; fusible metal is more rigid to swage upon than tin. The die metal is an alloy of tin, twenty-four ounces; antimony, three ounces; copper, one and one-half ounces; zinc, one ounce; phosphor tin, one-fourth ounce. This metal is similar to Babbett, but much better. A piece of the sheet composite metal is cut to pattern and swaged upon the metal model in the shot-swager. Owing to its softness and pliability, a plate is easily and accurately swaged, taking only a few minutes. The gold side will, of course, be next the model. After the plate is swaged it has to be punched with slots around the ridge. The slots correspond to the position of wire attachments for a gold plate with the teeth mounted in vulcanite. The perforations are also made across the plate close to the posterior edge of the palate for an upper denture. I have designed a special punch for making these perforations; it acts by throwing up two barbs and curling them over at each end of the perforation, making a very strong anchorage for the vulcanite. The punch may with advantage be used for securing vulcanite to ordinary gold plates, and save the time and trouble of fitting wire attachments in the usual manner. The slots may be made of different sizes by adjusting the screw fitted to the handle. In perforating the plate it should be held loosely in the hand to prevent warping. If this should happen, however, it is quickly corrected by swaging again with fine shot, which does not injure the barbs thrown up by the punch, as would occur with any other method of swaging. The subsequent process of combining these strengtheners with vulcanite in the plaster mould of flask is the same as with an ordinary gold plate, with the exception that the pattern for the mould for the palate is made of swaged sheet tin sufficiently thick for the vulcanite when taking its place to cover the silver or lingual side of the plate, the gold lining forming the palatal side.

The weight of a medium size plate of this composite metal for an upper edentulous case is about four to five pennyweights, and the cost, at forty shillings per ounce, will be about eight shillings. I have been making this work for some time with much satisfaction, and have a number of cases in use. The advantages are a pure gold surface in contact with the gums, a strengthener to the vulcanite, a more accurate fit than with an ordinary gold plate, and the easy and ready method of swaging the plate and making the perforations to combine it with the vulcanite. I am trying this metal now by covering both sides of the dental alloy with pure gold and rolling to No. 6 or 7 gauge for use as an ordinary gold plate with rubber attachments. The metal is easier and more accurately swaged than 18- or 20-carat gold plate, and keeps the pure gold color in the mouth under all conditions, and the layer of gold is so strong prepared in the manner described that I expect it to wear any length of time. As it can be supplied by the depots at fifty shillings per ounce, a plate will cost considerably less than ordinary gold plate at from seventy to eighty shillings per ounce. For lining and strengthening vulcanite cases as first described there is, without doubt, a future before it.—*Journal of the British Dental Association.*

